# Hints

**Published:** 1884-07

**Authors:** S. O. Gleason


### HINTS.

S. O. GLEASON, M. D.

Every man by virtue of birth into this life, comes into possession of a given amount of brain and muscular force or energy. There is a possibility of doing just so much, no more. The great practical question of life is—how best to utilize the sum total of inherited energy or power.

There can be no question but that the brain and muscular systems—should have each a regular training—so as to secure a well-balanced organism. There should be a special training for some regular business. Then all the forces—so trained—should be cared for as faithfully and carefully as one would his bank account. Being sure not to expend his energies, so inherited and trained, as to endanger them—or to rapidly exhaust his force by any forms of excess in habits of life.

It takes from twenty to thirty years to make a boy child into a man, who can command all his forces and use them in any emergency in the line of life for which he has been specially fitted. Such an one has cost—say, from five to ten thousand dollars. Now a man must take about one-half of his time to eat and sleep. Suppose then—though he works from thirty years of age until he is sixty—he has only fifteen years of active, wide-awake life.

Any one at a glance, can now see how carefully should any man be of his body—how religiously he should care for and conserve his energies and not exhaust his force by any habits of life that he can avoid.

Professional—hard-working men, never forget the rich treasure entrusted to you in your bodies. Give them good and kindly care and you will receive a rich reward in a long life of usefulness and pleasure. Nature everywhere asserts the law of rest, repose, quiet. During the long winter all forms of vegetable life rest.

Each twenty-four hours of time has its night and its day. Let us see to it that we give ourselves all the rest we need. We must not expend more force each day than we can make up by food and rest. Even engines—it has been found—last better and can in the long run, perform more work by short runs and frequent periods of rest.

It is sad—one of the saddest things that can come to a well-trained man—to use up and exhaust his splendid forces before he is—at least sixty to seventy years old.

I know men and love them who are bright and useful at seventy. They are splendid specimens of manhood—rich in knowledge and experience—a comfort to all who meet them.

Men in these days of knowledge find so much to learn—so much to do—that they need to shut off steam and put on the breaks very often, else they will come to disaster and fail of the real comforts of a long life.

We all come to the time in life when great care is absolutely necessary. We must hear the voice of nature—rest more and work less—remembering that with advancing years our limitations increase.

